# Dysregulation of ACE-1 in Normal Aging and the Early Stages of Alzheimer’s Disease

**DOI:** 10.1093/gerona/glac083

**Published:** 2022-04-09

**Authors:** Robert MacLachlan, Patrick Gavin Kehoe, J Scott Miners

**Affiliations:** Dementia Research Group, Clinical Neurosciences, Bristol Medical School, University of Bristol, Learning and Research Building, Southmead Hospital, Bristol, UK; Dementia Research Group, Clinical Neurosciences, Bristol Medical School, University of Bristol, Learning and Research Building, Southmead Hospital, Bristol, UK; Dementia Research Group, Clinical Neurosciences, Bristol Medical School, University of Bristol, Learning and Research Building, Southmead Hospital, Bristol, UK

**Keywords:** Aging, Alzheimer’s disease, Angiotensin-converting enzyme-1, Angiotensin-converting enzyme-2, Angiotensin-II

## Abstract

An imbalance in the renin–angiotensin system (RAS) is associated with cognitive decline and disease pathology in Alzheimer’s disease (AD). In this study, we have investigated changes in the brain angiotensin-converting enzyme-1 (ACE-1) and angiotensin-II (Ang-II), and the counter-regulatory angiotensin-converting enzyme-2 (ACE-2), in the frontal and temporal cortex during normal aging and in the early stages of AD. We studied a cohort of normal aging (*n* = 121; 19–95 years age-at-death) from the Sudden Death Brain Bank, University of Edinburgh, United Kingdom, and AD and age-matched controls (*n* = 60) from the South West Dementia Brain Bank, University of Bristol, United Kingdom, stratified according to Braak tangle stage (BS): 0–II, III–IV (intermediate disease), and V–VI (end-stage disease). ACE-1 and ACE-2 enzyme activity were measured using fluorogenic peptide activity assays. ACE-1, ACE-2, and Ang-II protein level were measured by enzyme-linked immunosorbent assay (ELISA). In both regions, ACE-1 protein and Ang-II levels correlated positively with age whereas ACE-1 enzyme activity was inversely related to age. ACE-1 protein correlated positively with Ang-II, whilst ACE-1 activity correlated inversely with Ang-II in normal aging. ACE-1 enzyme activity was elevated at an early/intermediate stage, BS III–IV compared to BS 0–II in the temporal cortex in AD. ACE-2 protein and enzyme activity were unchanged with aging and in AD. In conclusion, ACE-1 activity is induced in the early stages of AD independently from normal physiological age-related changes in ACE-1 protein.

The renin–angiotensin system (RAS) is involved in the systemic and neurogenic regulation of blood pressure (1). Circulating angiotensin-II (Ang-II) can enter the brain in regions without a blood–brain barrier (BBB), such as the circumventricular organs, and interact with brain RAS receptors to regulate systemic blood pressure and maintain volume homeostasis (2). Brain RAS is also expressed within various brain stem and ventricular nuclei and may function independently from circulating RAS to regulate sympathetic outflow and cardiac activity (although it is unclear whether renin originates within the brain or is systemically-derived (3)). Nevertheless, there is an established literature indicating that RAS, particularly the enzymes, peptides, and receptors involved in mediating the downstream RAS pathways, are altered within the brain in aging, hypertension, and dementia (4).

Alzheimer’s disease (AD) is the most common cause of dementia accounting for approximately 60%–80% of all dementia cases. Aging is the most important risk factor for cognitive decline and the development of AD. AD is not a continuum of normal aging although studies indicate that normal aging and AD share common genetic, molecular, and cellular traits (5). Angiotensin-converting enzyme-1 (ACE-1), responsible for the generation of Ang-II is relatively highly expressed in the frontal and temporal lobe, including the hippocampus (6) and is altered in AD (7). Our own studies in human postmortem brain tissue have shown that the ACE-1/Ang-II generating pathway is overactive in the frontal cortex (FCx) in AD (8,9). A recent study showed higher Ang-II type 1 receptor (AGTR1) levels in the FCx in relation to cognitive decline and elevated markers of disease pathology, including raised levels of oxidative stress, inflammation, Aβ, and tau (10). We have also previously shown that the protective counter-regulatory pathways, including the ACE-2/Ang-(1–7) pathway (8,11), and aminopeptidase-mediated conversion of ANG-II to Ang-III/Ang-IV (12) are dysregulated in AD. The imbalance in the ACE-2/Ang-(1–7) pathway within the brain is strongly associated with higher levels of parenchymal beta-amyloid and tau load in AD (8,11,12).

AGTR1 blockers and ACE-1 inhibitors (reviewed by Kehoe et al. (13)), or experimental activation of the counter-regulatory RAS pathway (14), are protective against the onset of cognitive decline and disease pathology in rodent models of AD. Supporting data from large epidemiological and interventional studies in humans indicate that commonly prescribed RAS-targeting medication, particularly those that cross the BBB, such as perindopril and captopril, improves cognition, reduces the incidence, and delays the onset of AD (15). Clinical trials are underway to determine if RAS-modifying drugs can provide protection in AD patients (7,15). Together, these data indicate that the classical ACE-1/Ang-II pathway is dysregulated in AD and that restoring the balance of downstream ACE-1 (overactive) and ACE-2 (underactive), or the balance between AGTR1 and AGTR2 expression (16) within the brain, are potential targets for therapeutic intervention in AD (17).

We still have a limited understanding of the timing of ACE-1 and ACE-2 changes in normal aging and AD within the brain. Characterizing the timing of these changes in relation to normal aging and the onset of AD, will potentially inform when therapeutic intervention will be most effective. Studies to-date, largely performed in rodents and restricted to peripheral organs, indicate that there is an organ-specific age-related imbalance in local RAS pathways resulting in overactivation of downstream disease-associated RAS pathways, with concurrent loss of counter-regulatory RAS signaling, independent of systemic RAS (18–20). Furthermore, we know little about the change in ACE-1 and ACE-2 in relation to early or intermediate disease stages in AD—most neuropathological studies in human brain tissue have focused on end-stage disease (8,11,12,21,22).

In this study, we have measured the key downstream RAS mediating enzymes (ACE-1 and ACE-2, respectively) and Ang-II levels (as a key determinant of prevailing signaling routes in counter-regulatory RAS pathways following conversion to Ang-[1–7] or Ang-III/Ang-IV) in the FCx and temporal cortex (TCx) in a cohort of normal aging and a separate AD and age-matched control cohort that was grouped according to disease stage severity: controls (Braak tangle stage 0–II), intermediate stage (Braak tangle stage III–IV), and end-stage disease (Braak tangle stage V–VI). We hypothesized that an age-related imbalance in brain ACE-1 and ACE-2 activity is exacerbated in the early stages of AD in association with disease pathology in AD.

## Method

### Study Cohort

A cohort of normal aging cases (*n* = 101; 19–80 years age-at-death) was obtained from the Sudden Death Brain Bank, Edinburgh University, UK with local ethical approval (REC reference: 16/ES/0084). Cases had no pathological diagnosis of neurodegenerative disease. The cases were stratified into the following age groups: ≤45 years, 46–65 years, and >65 years for certain analyses.

AD cases and age-matched nondementia controls (*n* = 60; 67–97 years age-at-death) were obtained from the South West Dementia Brain Bank, University of Bristol, UK with local ethical approval (REC reference: 18/SW/0029). A neuropathological diagnosis of AD was made according to NIA-AA guidelines (23). Control cases had no history of dementia, a Braak tangle stage of III or less, with few or absent neuritic plaques, and no other neuropathological abnormalities apart from scattered diffuse plaques in some cases. The AD and age-matched control brain cohort was subdivided according to Braak tangle stage (0–II, III–IV, and V–VI; *n* = 20 per group). The demographic data and neuropathological findings and the MRC identifier numbers are summarized in Supplementary Tables 1–4.

### Brain Tissue

Brain tissue (200 mg) from the mid-FCx (Brodmann area 46) and TCx (superior temporal gyrus; Brodmann area 41/42), was homogenized in 1 mL of 1% SDS (sodium dodecyl sulfate) buffer with protease inhibitors in a Precellys 24 homogeniser (Bertin Technologies, Montigny-le-Bretonneux, France) as previously described (22). The samples were then centrifuged at 12,000*g* at 4°C, and the supernatant was aliquoted and stored at −80°C.

### ACE-1 Enzyme Activity Assay

ACE-1 activity was measured in brain tissue homogenates prepared in 1% SDS lysis buffer using an ACE-1 specific fluorogenic substrate (Abz-FRK(Dnp)-P) as described in previous studies (8,22). We previously showed that 1% SDS buffer does not negatively impact ACE-1 enzyme activity in brain tissue homogenates, compared to lysis buffers prepared without detergents, or prepared with commonly used nondenaturing detergents such as NP-40 and Triton-X100 (22). Brain homogenates were diluted 1:10 in HEPES assay buffer (50 µM HEPES (hydroxyethyl-1-piperazineethane sulfonic acid), pH 6.5). Recombinant human ACE-1 (R&D Systems, Abington, Oxford, UK) was diluted 2-fold in HEPES buffer to generate a reference standard curve (450–28 750 pg/mL). Ten microliters of captopril (10 µM; 1 mM stock diluted 1 in 100 in distilled water; Enzo Life Sciences, Exeter, UK) or distilled water was added to “inhibited” and “uninhibited” rows of samples and standards, respectively in a black Fluoronunc 96-well plate (Fisher Scientific, Loughborough, UK). Fifty microliters of sample, recombinant standard or blank (assay buffer alone) was added to the plate in triplicate (2 in uninhibited rows and 1 in an inhibited row) and incubated at 26°C for 10 minutes. Fifty microliters of Abz-FRK(Dnp)-P (Enzo Life Sciences), diluted 1 in 200 in HEPES assay buffer, was then added to each well. The plate was covered in foil, minimizing exposure to light, and incubated at 26°C for 2.5 hours. The level of fluorescence was read using a microplate reader (FLUOstar OPTIMA; BMG Labtech, Aylesbury, UK) at excitation 320 nm and emission 405 nm. ACE-1 activity was calculated for each sample by subtracting the inhibited activity from the mean uninhibited activity and was expressed as relative fluorescence units (r.f.u).

### ACE-1 Protein ELISA

The concentration of ACE-1 protein was measured in brain tissue homogenates using a commercially available duoset ACE-1 enzyme-linked immunosorbent assay (ELISA) following manufacturer’s guidelines (R&D Systems) as in a previous study (22). In brief, the capture antibody was diluted in PBS (phosphate-buffered saline) (1 600 ng/mL) and incubated in a NUNC maxisorp 96-well plate (R&D Systems) overnight at room temperature. The plate was washed 5 times in 0.5% PBS:Tween-20, and then blocked with 1% BSA (bovine serum albumin):PBS for 1 hour at room temperature. After washing a further 5 times, brain homogenates diluted in 1% BSA:PBS (1/80) and a serial dilution of recombinant ACE-1 to generate a standard curve (125–8 000 pg/mL) was added to respective wells for 2 hours at room temperature. After a further wash step, detection antibody, diluted in 1% BSA:PBS (400 ng/mL), was added to the plate and incubated for 2 hours at room temperature. The plate was washed again, and streptavidin horse-radish peroxidase (HRP) (1/200 in PBS:0.01% Tween-20) was added for 20 minutes at room temperature in the dark. After a final wash step, TMB (tetramethylbenzidine) substrate (R&D Systems) was added to each well for 30 minutes at room temperature, and 2N sulphuric acid was added to stop the reaction. Absorbance was read at 450 nm using a microplate reader (FLUOstar OPTIMA; BMG Labtech). The concentration of ACE-1 in each sample was interpolated from the reference standard curve. Each sample was measured in duplicate and the average calculated.

### ACE-2 Enzyme Activity Assay

ACE-2 activity was measured in brain tissue homogenates using the ACE-2 specific fluorogenic substrate (Mca-APK(Dnp)), as described in our previous study (11). Brain homogenates were diluted 1:10 in Tris assay buffer (75 mM Tris, 1 M NaCl, pH 7.5), and recombinant human ACE-2 (R&D Systems; 20–1 250 pg/mL) was diluted 2-fold in assay buffer to create a reference standard curve for each plate. Ten microliters of distilled water or an ACE-2 inhibitor (MLN-4760, diluted to 2 µg/mL in distilled water; Millipore, Darmstadt, Germany) was added to the uninhibited and inhibited rows, respectively in a black Fluoronunc 96-well plate (Fisher Scientific). Fifty microliters of sample, standard or blank were added to respective wells in triplicate (2 in uninhibited wells and 1 in an inhibited well) and incubated at 37°C for 10 minutes. Fifty microliters of Mca-APK(Dnp), diluted 10 µg/mL in assay buffer (Enzo Life Sciences), was added to each well. The plate was covered in foil minimizing exposure to light and incubated in a plate shaker at 37°C for 3 hours. The level of fluorescence was read using a microplate reader (FLUOstar OPTIMA; BMG Labtech) at excitation 330 nm and emission 390 nm. ACE-2 activity was calculated for each sample by subtracting the inhibited activity from the mean uninhibited activity and presented as r.f.u.

### ACE-2 Protein ELISA

The concentration of ACE-2 protein was measured in brain tissue homogenates using a commercially available ACE-2 ELISA duoset (R&D Systems) following manufacturer’s guidelines with the following modifications: capture antibody was used at 4 µg/mL (2-fold higher than recommended); detection antibody at 200 ng/mL (2-fold higher than recommended); and strep:HRP at 1 in 20 (2-fold higher than recommended). Brain tissue homogenates were diluted 1 in 10 in PBS:1% BSA, and a serial dilution of recombinant ACE-2 (40–2 500 pg/mL) was used to generate a standard curve. The timings for each step and the wash steps were the same as described earlier for the ACE-1 duoset ELISA. TMB substrate (R&D Systems) was added to each well for 30 minutes at room temperature, and 2N sulphuric acid was added to stop the reaction. Absorbance was read at 450 nm for each well using a microplate reader (FLUOstar OPTIMA; BMG Labtech). The concentration of ACE-2 in each sample was measured in duplicate, and the average was calculated after interpolation from the standard curve.

### Ang-II ELISA

Ang-II concentration was measured by direct ELISA using an in-house assay as previously described (14,24). Brain homogenates were diluted in PBS (1:20) and incubated alongside a serial dilution of Ang-II (Abcam, Cambridge, UK; 78–5 000 ng/mL) in a NUNC maxisorp 96-well plate (R&D Systems) for 2 hours with shaking at room temperature. The plate was washed 5 times in 0.5% Tween-20:PBS, blocked with 1% BSA:PBS for 1 hour at room temperature, washed again as above and a biotinylated antibody specific for Ang-II (1 µg/mL; Cloud-Clone, Wuhan, China) was incubated for 2 hours. After a further wash step, streptavidin horse-radish peroxidase (1:200, R&D Systems) was added for 20 minutes at room temperature, the plate was further washed, and TMB substrate (R&D Systems) was added for 30 minutes at room temperature. 2N sulphuric acid was added to stop the color change. Absorbance was read using a microplate reader (FLUOstar OPTIMA; BMG Labtech) at 450 nm for each well. Ang-II concentration was interpolated from the standard curve, and each sample was measured in duplicate and the average calculated.

### Statistical Analysis

Unpaired *t* tests, ANOVA with Tukey’s post hoc analysis, or Kruskal Wallis analysis, was used for comparisons between grouped data where appropriate. Spearman’s rank order was used to assess correlation. GraphPad Prism version 8 was used for statistical analyses. p Values <.05 were considered to be statistically significant.

## Results

### ACE-1 and Ang-II Protein Level Were Increased With Normal Aging, Whereas ACE-1 Activity Declined

**Figure 1.**
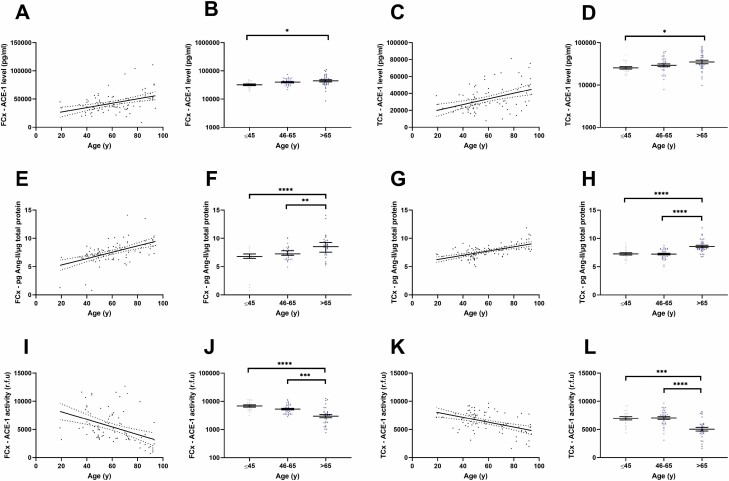
ACE-1 and Ang-II protein levels are elevated in relation to normal aging in the frontal and temporal cortex. (**A–D**) Scatterplots showing a positive correlation between ACE-1 protein concentration and age in the frontal cortex (FCx―*n* = 84; *r* = 0.362, *p* = .0006) and temporal cortex (TCx―*n* = 84; *r* = 0.408, *p* = .0001). ACE-1 protein was elevated in those aged 65 years and older compared to those aged ≤45 years in the FCx (≤45 years *n* = 18, 46–65 years *n* = 35, >65 years *n* = 31; *p* = .012) and TCx (≤45 years *n* = 18, 46–65 years *n* = 35, >65 years *n* = 31; *p* = .039). (**E–H**) Scatterplots showing a positive correlation between Ang-II concentration normalized to the total protein and age (FCx—*n* = 84; *r* = 0.530, *p* < .0001 and TCx— *n* = 84; *r* = 0.559, *p* < .0001). A significant increase in Ang-II concentration in those aged 65 years and above was observed across both regions (FCx―≤45 years *n* = 18, 46–65 years *n* = 35, >65 years *n* = 31; ≤45 years vs >65 years *p* < .0001, 46–65 years vs >65 years *p* = .0057 and TCx―≤45 years *n* = 18, 46–65 years *n* = 35, >65 years *n* = 31; ≤45 years vs >65 years *p* < .0001, 46–65 years vs >65 years *p* < .0001). (**I–L**) Scatterplots showing an inverse correlation between ACE-1 enzyme activity and age (FCx—*n* = 84; *r* = −0.520, *p* < .0001 and TCx—*n* = 84, *r* = −0.464, *p* < .0001) and a highly significant reduction in ACE-1 activity in those aged 65 years and older (FCx―≤45 years *n* = 18, 46–65 years *n* = 35, >65 years *n* = 31; ≤45 years vs >65 years *p* < .0001, 45–65 years vs >65 years *p* = .0001 and TCx―≤45 years *n* = 18, 46–65 years *n* = 35, >65 years *n* = 31; ≤45 years vs >65 years *p* = .0001, 46–65 years vs >65 years *p* < .0001). ACE-1 activity is expressed as relative fluorescence units (r.f.u). In A, C, E, G, I, and K, linear regression lines and 95% confidence interval lines are shown. In B, D, F, H, J, and L, standard error of the mean bars is shown. *p < .05, **p < .01, ***p < .001, and ****p < .0001. ACE-1 = angiotensin-converting enzyme-1; Ang-II = angiotensin-II.

ACE-1 protein level correlated positively with age within the FCx (Spearman correlation; *r* = 0.362, *p* = .0006; Figure 1A) and TCx (Spearman correlation; *r* = 0.408, *p* = .0001; Figure 1C). When stratified into age groups (<45 years, 45–65 years, and >65 years), ACE-1 level was significantly elevated in those aged 65 years and older in both the FCx and TCx (Figure 1B and D).

Ang-II protein level correlated positively with age in the FCx (Spearman correlation; *r* = 0.530, *p* < .0001; Figure 1E) and TCx (Spearman correlation; *r* = 0.559, *p* < .0001; Figure 1G). Ang-II protein was similarly elevated in those aged 65 years and older across both regions (Figure 1F and H).

ACE-1 enzyme activity, in contrast, was negatively correlated with age in the FCx, (Spearman correlation; *r* = −0.520, *p* < .0001; Figure 1I) and TCx (Spearman correlation; *r* = −0.464, *p* < .0001; Figure 1K). ACE-1 activity was lower in those aged 65 years and older compared to ≤45 years and the 45–65 years age group in the FCx (Tukey’s; ≤45 years vs 46–65 years *p* = .23, ≤45 years vs >65 years *p* < .0001, 45–65 years vs >65 years *p* = .0001; Figure 1J). A similar pattern was evident in the TCx (Tukey’s; ≤45 years vs 46–65 years *p* = .99, ≤45 years vs >65 years *p* = .0001, 46–65 years vs >65 years *p* < .0001; Figure 1L).

**Figure 2.**
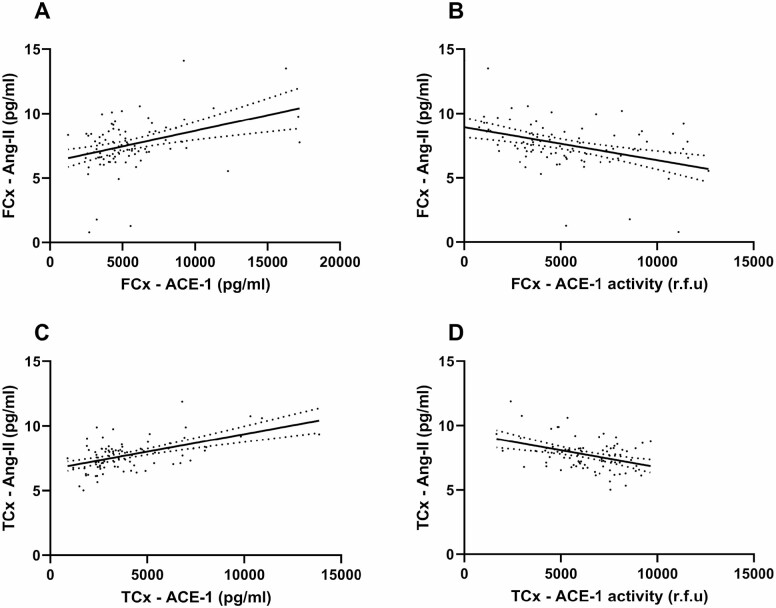
ACE-1 protein level is positively correlated, and ACE-1 enzyme activity is negatively correlated, with Ang-II in normal aging. (**A** and **C**) Scatterplots showing a positive correlation between ACE-1 protein level and Ang-II concentration in the frontal cortex (FCx―*n* = 87; *r* = 0.290, *p* = .0065) and temporal cortex (TCx―*n* = 85; *r* = 0.452, *p* < .0001). (**B** and **D**) Scatterplots showing an inverse correlation between ACE-1 enzyme activity and Ang-II concentration in both regions (FCx—*n* = 87; *r* = −0.391, *p* = .0002 and TCx—*n* = 84; *r* = −0.278, *p* = .011). ACE-1 activity is expressed as relative fluorescence units (r.f.u). Linear regression lines of best fit and 95% confidence intervals are shown. ACE-1 = angiotensin-converting enzyme-1; Ang-II = angiotensin-II.

Ang-II concentration correlated positively with ACE-1 protein levels in both the FCx (Spearman correlation; *r* = 0.290, *p* = .0065) and TCx (Spearman correlation; *r* = 0.452, *p* < .0001) and negatively with ACE-1 enzyme activity across both regions (Spearman correlation; FCx—*r* = −0.391, *p* = .0002; TCx—*r* = −0.278, *p* = .011; Figure 2A–D).

### ACE-2 Protein Concentration and Activity Were Unaltered With Aging

In the FCx and TCx, neither ACE-2 protein level nor enzyme activity changed with age (Supplementary Figure 1). ACE-2 protein level correlated positively with Ang-II level in the FCx (Spearman correlation; *r* = 0.308, *p* = .0037) and TCx (Spearman correlation; *r* = 0.409, *p* = .0001). No correlations were observed between ACE-2 enzyme activity and Ang-II concentrations in either brain region (Supplementary Figure 2).

### The Ratio of ACE-1:ACE-2 Protein Increased in Normal Aging Whereas the Ratio of ACE-1:ACE-2 Enzyme Activity Declined

**Figure 3.**
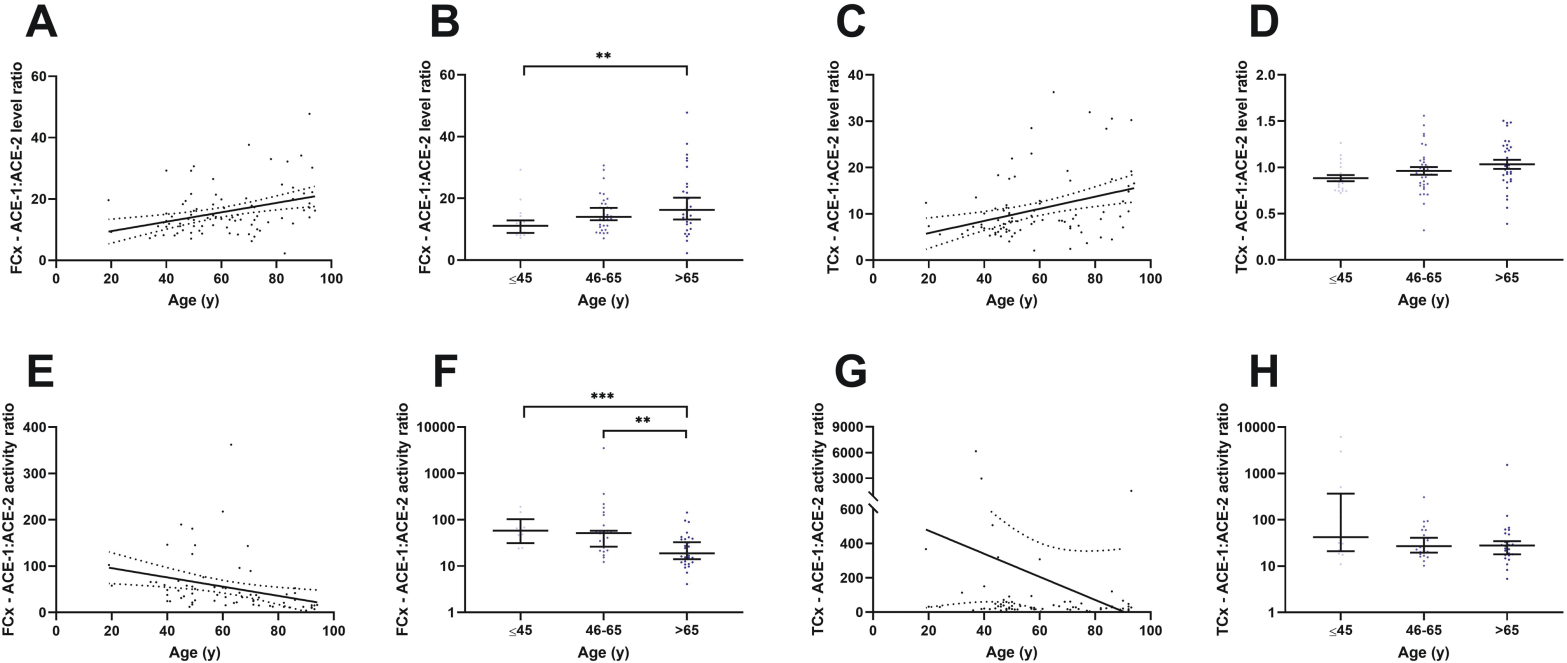
The ratio of ACE-1:ACE-2 protein concentration is increased with normal aging in contrast to enzyme activity. (**A–D**) Scatterplots showing a positive correlation between the ratio of ACE-1:ACE-2 protein concentration and age in the frontal cortex (FCx―*n* = 87; *r* = 0.356, *p* = .0007) and temporal cortex (TCx―*n* = 86; *r* = 0.358, *p* = .0007). The ratio of ACE-1:ACE-2 protein concentration was significantly elevated in those aged >65 years and older compared to ≤45 years in the FCx (≤45 years *n* = 18, 46–65 years *n* = 35, >65 years *n* = 34; *p* = .0073). No change was observed in the TCx (≤45 years *n* = 20, 46–65 years *n* = 35, >65 years *n* = 31). (**E–H**) Scatterplots showing an inverse correlation between the ACE-1:ACE-2 enzyme activity ratio and age (*n* = 69; *r* = −0.587, *p* < .0001) and a reduction in the ACE-1:ACE-2 enzyme activity ratio in the 65 years and older age group in the FCx (≤45 years *n* = 14, 46–65 years *n* = 26, >65 years *n* = 30; ≤45 years vs >65 years *p* = .0009, 45–65 years vs >65 years *p* = .0023). No differences were observed in the TCx. Linear regression line of best fit and 95% confidence intervals and medians with 95% confidence intervals are shown. **p < .01 and ***p < .001. ACE-1 = angiotensin-converting enzyme-1; ACE-2 = angiotensin-converting enzyme-2.

The ratio of ACE-1:ACE-2 protein level correlated positively with age in both the FCx and TCx (Spearman correlation; FCx: *r* = 0.356, *p* = .0007; TCx: *r* = 0.358, *p* = .0007; Figure 3A and C). Stratification into age groups showed a significant increase in those aged 65 years and older compared to the ≤45 years group in FCx (*p* = .0073; Figure 3B) which was not observed in the TCx (Figure 3D).

The ratio of ACE-1:ACE-2 enzyme activity, in contrast, was inversely correlated with age in the FCx (Spearman correlation; *r* = −0.587, *p* < .0001; Figure 3E) which, upon stratification into age groups, was significantly reduced in the 65 years and older compared to ≤45 years group (*p* < .001) and 46–65 years age group (*p* < .01; Figure 3F). In contrast, no significant differences were observed in the TCx (Figure 3G–H).

### ACE-1 activity increased in the early stages of AD whereas ACE-1 activity and Ang-II level were unchanged

We next investigated changes in ACE-1, ACE-2, and Ang-II in a separate AD and age-matched control cohort that was stratified according to Braak tangle stage to assess changes in relation to disease stage.

**Figure 4.**
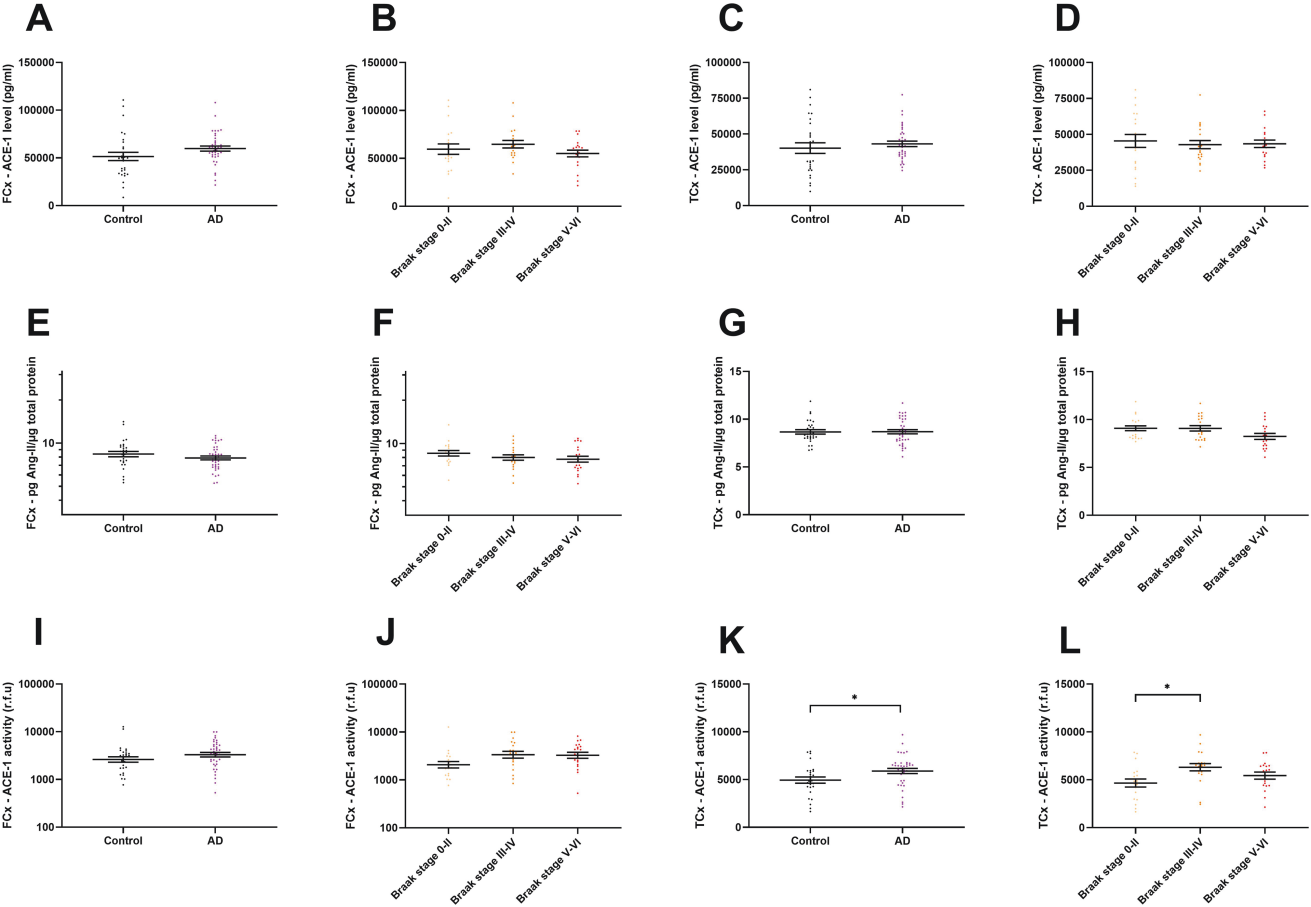
ACE-1 enzyme activity is increased in the early stages of Alzheimer’s disease (AD). (**A–D**) Scatterplots showing ACE-1 protein level is unchanged between AD and age-matched controls (control = 27, AD = 39), and in relation to Braak tangle stage (BS 0–II = 18, BS III–IV = 18, BS V–VI = 19), in the frontal cortex (FCx) and temporal cortex (TCx). (**E–H**) Scatterplots showing no change in Ang-II concentration, normalized to the total protein, between AD and age-matched controls (control = 27, AD = 39) and Braak tangle stage in the FCx and TCx. (**I–L**) Scatterplots showing no significant differences in ACE-1 activity between control and AD cases (control = 27, AD = 39) and BS (BS 0–II = 18, BS III–IV = 18, BS V–VI = 19) in the FCx and a significant increase in ACE-1 activity in AD compared to age-matched controls (control = 27, AD = 38; *p* = .028) and in BS III–IV compared to BS 0–II (BS 0–II = 19, BS III–IV = 20, BS V–VI = 18; *p* = .01) in the TCx. ACE-1 activity is expressed as relative fluorescence units (r.f.u). Standard error of the mean is shown. *p < .05. ACE-1 = angiotensin-converting enzyme-1; Ang-II = angiotensin-II.

ACE-1 protein level was unchanged in AD (unpaired *t* test; *p* = .11; Figure 4A) and did not vary according to Braak tangle stage (BS; Tukey’s; BS 0–II vs BS III–IV *p* = .69, BS III–IV vs V–VI *p* = .28) in the FCx (Figure 4B). Similarly, in the TCx, no significant difference in ACE-1 level was seen between AD and age-matched controls (unpaired *t* test; *p* = .48; Figure 4C) or with Braak tangle stage (Tukey’s; BS 0–II vs III–IV *p* = .86, BS III–IV vs V–VI *p* = .99; Figure 4D).

In both the FCx and TCx, Ang-II was also unchanged in AD compared to controls (unpaired *t* test; *p* = .24 and unpaired *t* test; *p* = .96, repsectively; Figure 4E and G) and was unaltered in relation to Braak tangle stage (Tukey’s; BS 0–II vs III–IV *p* = .55, BS III–IV vs V–VI *p* = .89 and Tukey’s; BS 0–II vs III–IV *p* = 1.0, BS III–IV vs V–VI *p* = .10, respectively; Figure 4F and H).

ACE-1 activity was unchanged in the FCx of AD compared to controls (unpaired *t* test; *p* = .17; Figure 4I) although a trend towards higher activity was observed in BS III–IV and BS V–VI compared to BS 0–II (Tukey’s; BS 0–II vs BS III–IV *p* = .09, BS 0–II vs BS V–VI *p* = .10; Figure 4J). In the TCx, ACE-1 activity was significantly increased in AD (unpaired *t* test; *p* = .028; Figure 4K) and was significantly elevated in BS III–IV compared to BS 0–II (Tukey’s; *p* = .01; Figure 4L) but no significant difference was observed between BS 0–II and V–VI (Tukey’s; *p* = .35) or between III–IV and V–VI (Tukey’s; *p* = .26; Figure 4L).

### ACE-2 Protein Level and Activity Were Unchanged in AD

ACE-2 concentration and enzyme activity was unaltered in AD and did not vary with Braak tangle stage across both regions (Supplementary Figure 3).

### ACE-1:ACE-2 Enzyme Activity Ratio Increased With Disease Whereas the ACE-1:ACE-2 Protein Level Ratio Was Unchanged

**Figure 5.**
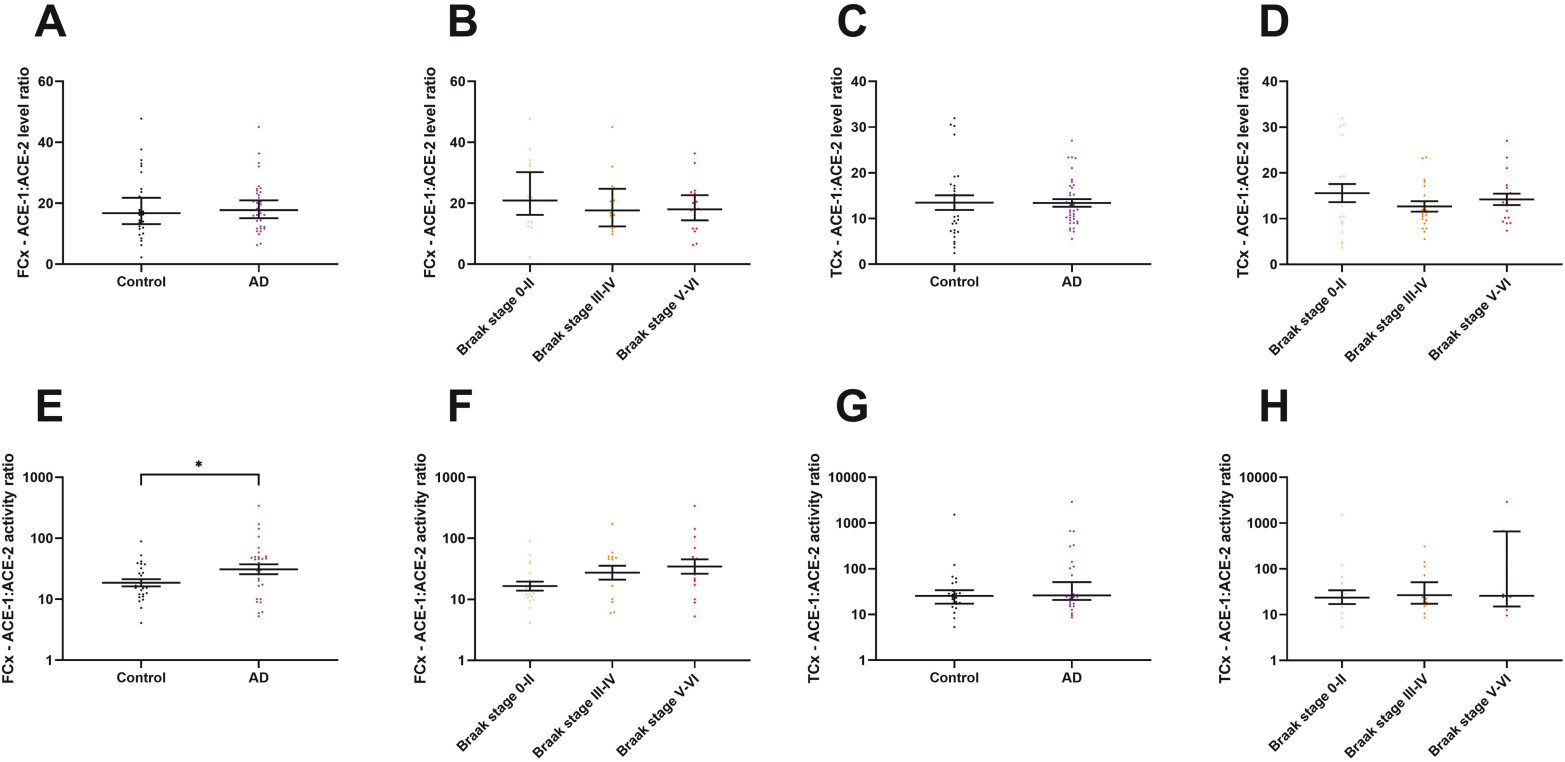
The ratio of ACE-1:ACE-2 enzyme activity is increased in Alzheimer’s disease (AD). (**A–D**) Scatterplots showing no change in ratio of ACE-1:ACE-2 protein concentration in relation to AD (control = 30, AD = 39) and Braak tangle stage (BS 0–II = 20, BS III–IV = 19, BS V–VI = 20) in the frontal cortex (FCx) and temporal cortex (TCx). (**E–H**) Scatterplots showing an increase in the ratio of ACE-1:ACE-2 enzyme activity between AD and controls (control = 26, AD = 30; *p* = .036) and a trend towards a higher ratio in relation to Braak stage (BS 0–II = 19, BS III–IV = 14, BS V–VI = 16) in the FCx. No change observed between AD and controls and BS in the TCx. Median and 95% confidence intervals are shown. *p < .05. ACE-1 = angiotensin-converting enzyme-1; ACE-2 = angiotensin-converting enzyme-2.

The ratio of ACE-1:ACE-2 protein level was unchanged in both the frontal and temporal regions in AD with no differences observed upon stratification into Braak tangle stage (Figure 5A–D). However, a significant increase in the ratio of ratio of ACE-1:ACE-2 enzyme activity can be seen in AD in the FCx (*p* = .036; Figure 5E), although no relationship was observed in relation to Braak tangle stage (Figure 5F). No significance differences were seen in the TCx (Figure 5G–H).

## Discussion

In this study, we have characterized age- and disease-related changes in ACE-1, ACE-2, and Ang-II in the FCx and TCx in AD. We have shown that local expression of ACE-1 and Ang-II protein levels, which correlated positively with each other, were both elevated with normal aging and rose significantly in individuals aged 65 years and older. In contrast, ACE-1 enzyme activity was reduced with normal aging and inversely related to Ang-II level. The ratio of ACE-1:ACE-2 protein level was increased in normal aging whereas the ratio of ACE-1:ACE-2 enzyme activity was reduced. In AD, ACE-1 enzyme activity was elevated specifically in the TCx and was highest in Braak tangle stage III–IV cases, that is, at an early/intermediate stage of disease. ACE-1 protein and Ang-II levels remained unchanged. The ratio of ACE-1:ACE-2 protein levels were unchanged but the ratio of ACE-1:ACE-2 enzyme activity was elevated in AD (opposite to normal aging). Together, these data indicate that ACE-1 protein and Ang-II levels are elevated within the FCx and TCx in normal aging but that these changes differ from AD in which ACE-1 enzyme activity becomes overactivated in the early stages of disease.

Systemic RAS, primarily known for regulating blood pressure, is often elevated in diseases involving the cardiovascular system and target organs. Locally expressed RAS pathways function both synergistically and independently from systemic RAS (2). It remains unclear whether renin, responsible for the conversion of angiotensinogen to angiotensin-I, is expressed locally within the brain. A recent study indicated that most brain renin originates in the bloodstream (3). Chymases, such as tonin and cathepsin D can produce angiotensin-I independently of renin within the brain (25,26). Further studies are necessary to confirm whether renin is expressed locally within the brain and to characterize disease-related changes in the upstream RAS pathway in AD. We have previously shown that downstream brain RAS pathways in the FCx are disturbed in AD—ACE-1 activity and Ang-II levels are elevated in AD, as are Ang-III levels (12), whereas ACE-2 activity is significantly reduced in AD (11). An imbalance in these downstream brain RAS pathways is related to disease pathology (Aβ and tau) in AD (8,11,22).

Despite age being a major contributor to cognitive decline and disease pathology in AD, we have only limited information regarding age-related changes in brain ACE-1 and ACE-2. Most studies to-date indicate that systemic RAS activity declines with age. Life-long therapeutic blockade of systemic cRAS has been shown to promote longevity in rodents (27,28). In contrast, organ-specific RAS pathways become overactive with age (20,29,30) indicating a shift towards hyperactive RAS—increased ACE-1, Ang-II, AGTR1 expression and loss of counter-regulatory protective RAS signaling, for example, lower ACE-2, AGTR2, and Mas receptor expression. Most of these studies, however, have been performed in peripheral organs including the lung, intestine, and vascular system (20,30–33), and it is unclear if the same pattern occurs in brain tissue in a human setting.

In this human postmortem study, we found that ACE-1 and Ang-II protein levels were elevated in relation to normal aging in the FCx and TCx, and ACE-1 protein correlated positively with Ang-II. In vitro studies, in human kidney tubular cells and in mouse neuronal cells, indicate that Ang-II can up-regulate the gene expression and protein concentration of renin (34) and ACE-1 (35,36) (and concurrently down-regulate ACE-2 gene and protein expression) thereby promoting overactivation of RAS. Unexpectedly, ACE-1 enzyme activity declined with age and correlated inversely with Ang-II concentration in our study. Previous studies have shown that intravenous (i.v.) infusion of Ang-II negatively regulates ACE-1 enzyme activity in the lung and testis in spontaneously hypertensive rats (37). Our observations in human brain tissue, are consistent with these in vitro findings, indicating that a physiological compensatory mechanism that limits ACE-1 enzyme activity in response to RAS overactivation with normal aging. The discrepancy between protein concentration and enzyme activity requires independent validation in larger studies, but we have previously shown divergence between ACE-1 protein level and enzyme activity in the FCx and cerbrospinal fluid (CSF) in AD (22).

In contrast to normal aging, ACE-1 enzyme activity (not ACE-1 protein or Ang-II) was elevated in the TCx in AD, supporting our previous observations (8,22). For the first time, to the best of our knowledge, we report that ACE-1 enzyme activity was elevated within the TCx specifically at an intermediate stage of AD, that is, BS III–IV. ACE-1 protein and Ang-II concentration, in contrast to normal aging, were unchanged in AD, and the relationships between Ang-II and ACE-1 protein that we observed in the aging controls were not apparent in the AD cohort. Furthermore, the ratio of ACE-1:ACE-2 enzyme activity was elevated in AD whereas in normal aging it was reduced. It is unclear why ACE-1 activity is elevated in AD without concurrent changes in ACE-1 protein. Perhaps the normal age-related protective response (posited above) may become dysfunctional in AD, for instance in the presence of Aβ peptides. Alternatively, disease-associated post-translational modification, altered secretion, or changes in the concentrations of potential endogenous inhibitors of ACE-1, may account for disparities between ACE-1 protein and activity in AD (38). We did not observe disease-associated changes to ACE-2 activity in this study compared to our previous study (11). Using G*Power software, we confirmed that the cohort in this study was underpowered to detect changes in ACE-2 enzyme activity in relation to normal aging and AD.

Hypertension, specifically in midlife, is a highly prevalent but modifiable risk factor for age-related cognitive decline and dementia. Several recent large meta-analyses of clinical trial data revealed that management of hypertension prevented cognitive decline and lowered the risk of dementia (39–42). Dementia rates are commonly reported to be lower in association with RAS modulation, particularly AGTR1 receptor blockers, in large observational studies (43–45). RAS modifying drugs are also associated with lower conversion to AD and a slower rate of cognitive decline (46) and reduced neurofibrillary tangle pathology at autopsy (47). A recent clinical trial of losartan, a classical AGTR1-receptor blocker, did not reduce rates of brain atrophy or cognitive decline compared to placebo in patients with mild-moderate AD (13). In view of recent studies indicating that BBB penetrating RAS agents better preserve memory compared to nonpenetrant BBB (48,49), future clinical trials should focus on RAS modifiers known to cross the BBB.

The timing of intervention is likely to be a critical factor in determining the success of RAS-modulation in clinical trials in AD. This is the first study to offer insights into changes in brain ACE-1 protein level and enzyme activity in relation to age- and disease-stage in AD. Our data reveal an induction of ACE-1 activity in early-stage AD, that is, Braak stage III–IV that is distinct from normal age-related changes in ACE-1 protein and Ang-II levels. ACE-1 activity can be measured in ante-mortem CSF and is elevated in AD (24) raising the possibility that changes in CSF ACE-1 enzyme activity alongside established CSF biomarkers (Aβ/tau), or changes in vascular markers such as soluble platelet-derived growth factor receptor, will be useful to identify those patients for which early RAS-targeted intervention will be most effective.

In conclusion, the present study reports age-related increased expression of ACE-1 and Ang-II protein concentrations, with a corresponding reduction in ACE-1 enzyme activity, possibly to limit further RAS activation in normal aging. In contrast, ACE-1 enzyme activity is elevated in AD, specifically at an intermediate stage of disease. Overall, these data indicate that brain RAS becomes dysregulated in the early stages of AD independently from age-related changes. The study improves our understanding of RAS in AD and may be important to inform the design and timing of future RAS-targeting clinical trials.

## Supplementary Material

glac083_suppl_Supplementary_Material
