# Supplementary material for: Dysregulation of ACE-1 in Normal Aging and the Early Stages of Alzheimer’s Disease
Source: J Gerontol A Biol Sci Med Sci. 2022 Apr 9;77(9):1775–83. doi: 10.1093/gerona/glac083 (PMC9434468; doi:10.1093/gerona/glac083)
Supplement: glac083_suppl_Supplementary_Material [file glac083_suppl_supplementary_material.pdf]

Supplementary data

eTable 1 - Supplementary Table 1: Demographic summary of the normal ageing cohort sex obtained from the Edinburgh Sudden Death Brain Bank, University of Edinburgh, UK.

eTable 2 - Supplementary Table 2: Demographic summary of the Alzheimer's disease cohort obtained from the South West Dementia Brain Bank, University of Bristol, UK.

eTable 3 - Supplementary Table 3: MRC brain bank unique identifiers for the South West Dementia Brain Bank cases

eTable4 - Supplementary Table 4: Summary of MRC identifiers for tissue obtained from Edinburgh Brain and Tissue Bank, stratified by age group

Supplementary Figure 1: ACE-2 enzyme activity and protein concentration is not altered in relation to age

Supplementary Figure 2: Angiotensin II positively correlates with ACE-2 protein level in normal ageing

Supplementary Figure 3. ACE-2 protein level and enzyme activity is unaltered in Alzheimer's disease.

Supplementary Table 2: Demographic summary of the normal ageing cohort sex obtained from the Edinburgh Sudden Death Brain Bank, University of Edinburgh, UK.

**Brain region**		**Frontal cortex (BA46)**		**Temporal cortex (BA41/42)**	
**Total (n)**		71		65	
**Sex**		**Male**	**Female**	**Male**	**Female**
**Age-at-death (y)**	**≤ 45 (n)**	11	7	13	7
	**45 < x ≤ 65 (n)**	32	7	26	10
	**> 65 (n)**	12	5	9	3
**Post-mortem delay (SD)**		79(±25)		75(±24)	

Supplementary Table 2: Demographic summary of the Alzheimer's disease cohort obtained from the South West Dementia Brain Bank, University of Bristol, UK.

**Brain region**		**Frontal cortex (BA46)**		**Temporal cortex (BA41/42)**	
**Total (n)**		60		60	
**Sex**		**Male**	**Female**	**Male**	**Female**
**Braak stage**	**0-II (n)**	10	10	10	10
	**III-IV (n)**	10	10	10	10
	**V-VI (n)**	10	10	10	10
**Mean age-at-death (SD)**		84(±7)		84(±7)	
**Post-mortem delay (SD)**		44(±10)		44(±10)	

Supplementary Table 3: MRC brain bank unique identifiers for the South West Dementia Brain Bank cases

**MRC identifier**		
**Braak stage 0-II**	**Braak stage III-IV**	**Braak stage V-VI**
BBN006.30198	BBN_8989	BBN_14405
BBN_24337	BBN_9038	BBN_22622
BBN_24561	BBN_9217	BBN_24324
BBN_19627	BBN006.26095	BBN_4214
BBN_22625	BBN_24317	BBN_9303
BBN_24311	BBN_26011	BBN_9332
BBN_24319	BBN_4215	BBN_9377
BBN_24325	BBN_8968	BBN_9378
BBN_24332	BBN_9050	BBN_9435
BBN_25025	BBN_9078	BBN006.27017
BBN006.28893	BBN_9220	BBN_24563
BBN006.29018	BBN_9257	BBN_24895
BBN006.30165	BBN_9269	BBN_26015
BBN006.31488	BBN_9331	BBN_9268
BBN006.32529	BBN_9343	BBN_9293
BBN006.32544	BBN_9394	BBN_9346
BBN006.32578	BBN006.26344	BBN_9367
BBN006.32826	BBN006.29640	BBN_9401
BBN006.32845	BBN006.29894	BBN_9420
BBN006.34115	BBN006.31445	BBN_9421

Supplementary Table 4: Summary of MRC identifiers for tissue obtained from Edinburgh Brain and Tissue Bank, stratified by age group

MRC identifier			
≤ 45y	46y < x ≤ 65y		> 65y
1.26976	1.33613	1.29525	BBN_2506
BBN_2360	1.29085	BBN_15221	BBN_2564
BBN_2442	BBN_18393	BBN_2372	1.30147
BBN_2494	BBN_2435	BBN_2385	BBN_13410
1.30972	BBN_15223	1.3293	1.26309
BBN_2562	1.29526	BBN_2476	BBN_2496
BBN_2389	BBN_3770	BBN_24780	BBN_2504
1.2896	1.30169	BBN_24781	1.33639
1.28959	BBN_2575	1.29467	1.29824
1.29466	BBN_2536	1.31503	1.29882
BBN_2509	1.29693	1.29533	BBN_2489
1.30841	BBN_2542	BBN_15809	1.30916
1.26124	BBN_2493	BBN_2571	1.32548
BBN_19591	BBN_2531	1.29084	1.30178
BBN_23395	BBN_22628	BBN_2510	BBN_2572
1.29529	1.29531	1.30833	1.30081
BBN_20592	1.26797	BBN_24195	1.30208
BBN_14396	1.34244	BBN_2505	BBN_2485
BBN_3784	1.3014	BBN_2511	BBN_2540
BBN_2495	BBN_25751	BBN_16425	BBN_14395
1.26313	BBN_2487	BBN_22612	BBN_4174
BBN_2421	1.34215	BBN_2486	1.28415
BBN_25055	BBN_2436	BBN_2451	
BBN_14397	BBN_22630	1.31439	
BBN_15222	BBN_4176	BBN_2550	
	1.28563	BBN_2513	
	1.31054	BBN_24479	

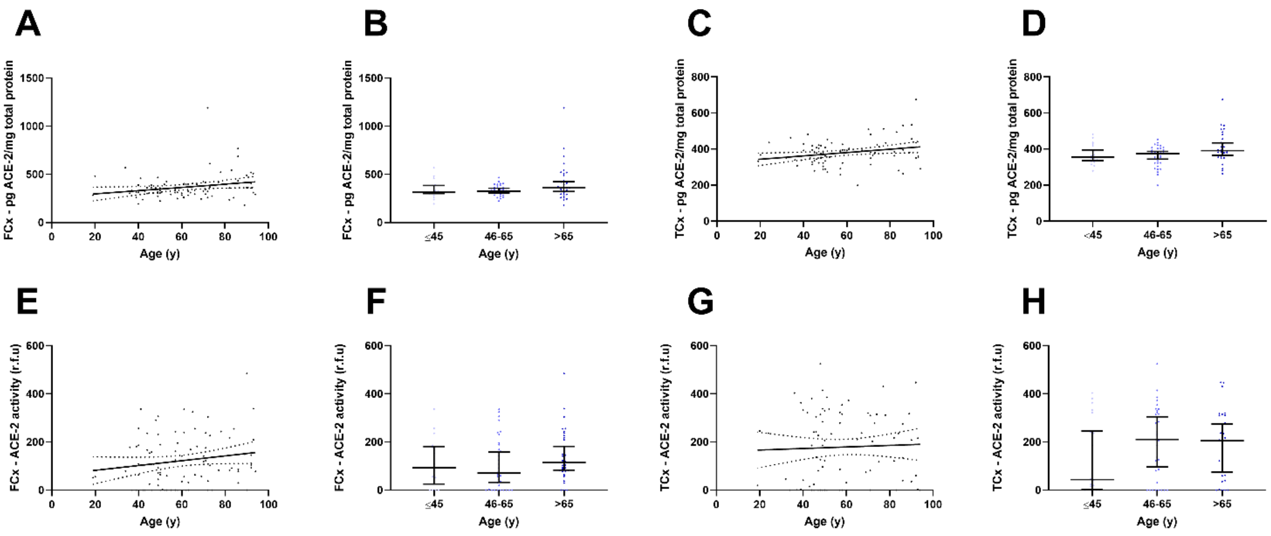

Supplementary Figure 1: ACE-2 enzyme activity and protein concentration is not altered in relation to age. **A-B**. Scatterplots showing no change in ACE-2 concentration, normalised to the total protein, in relation to age in the frontal cortex (FCx) (n=84; p=0.0651) or when stratified into the following age groups: ≤45 n=18, 46-65 n=35, >65 n=31 (p=0.0954). **C-D**. Scatterplots showing that ACE-2 concentration varied between age-groups (p=0.04923) however, no significant differences were found by *post-hoc* analysis in the temporal cortex (TCx). **E-F**. Scatterplot showing no correlation between ACE-2 activity and age (n=84; p=0.1493), or across different age groups (≤45y n=18, 46-65y n=35, >65y n=31; p=0.2326), in the frontal cortex (FCx) **G-H**. Scatterplots showing no correlation between ACE-2 activity and age (n=84; p=0.6592), or changes in ACE-2 activity in across different age-groups (≤45y n=18, 46-65y n=35, >65y n=31; p=0.5851), in the temporal cortex (TCx). ACE-2 activity is expressed as relative fluorescence units (r.f.u). A, C, E, G show linear regression lines with 95% confidence intervals. B, D show mean with standard error bars. F, H show median and 95% confidence intervals.

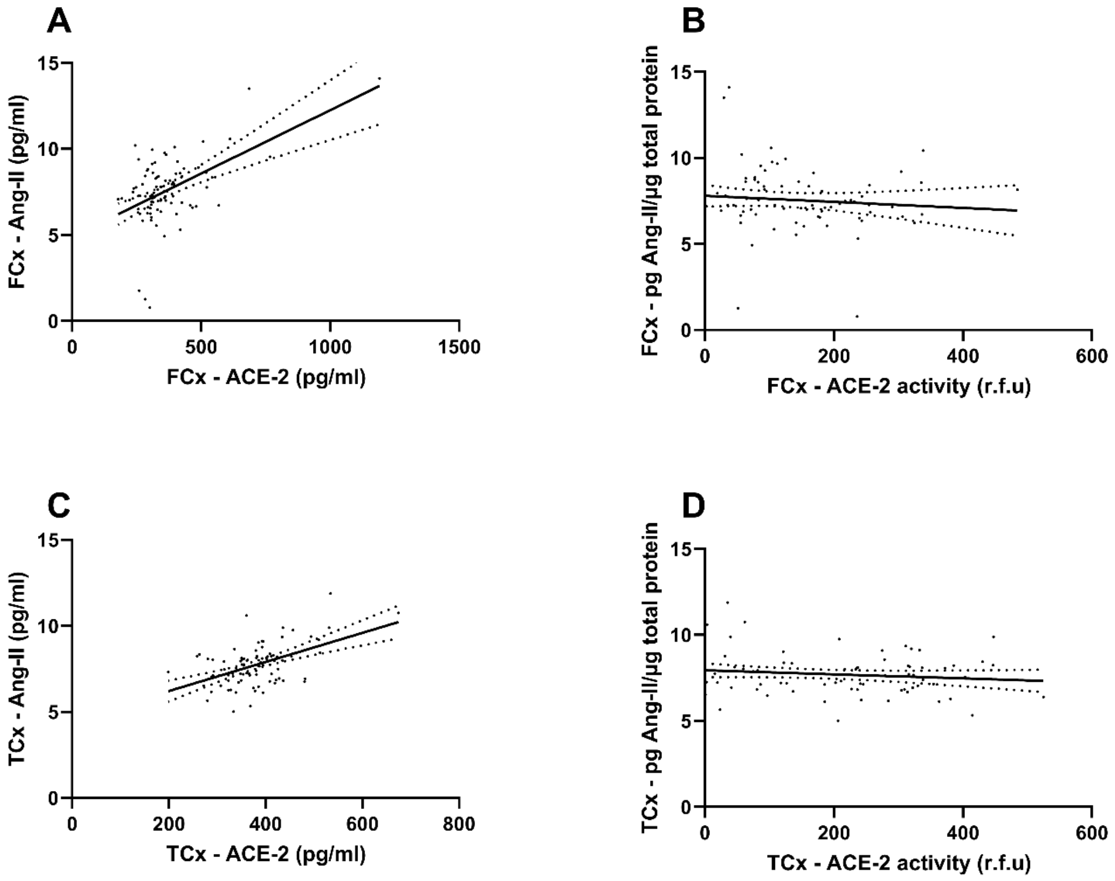

Supplementary Figure 2: Angiotensin II positively correlates with ACE-2 protein level in normal ageing. A and C. Scatterplots showing a positive correlation between Ang-II and ACE-2 protein concentration in the frontal (FCx) ($n=87$, $r=0.3081$, $p=0.0037$) and temporal cortex (TCx) ($n=85$, $r=0.4085$, $p=0.0001$). B and D. Scatterplots showing no relationship between Ang-II concentration and ACE-2 enzyme activity in the frontal and temporal cortex. ACE-2 activity is expressed as relative fluorescence units (r.f.u). Linear regression line of best fit and 95% confidence intervals are shown.

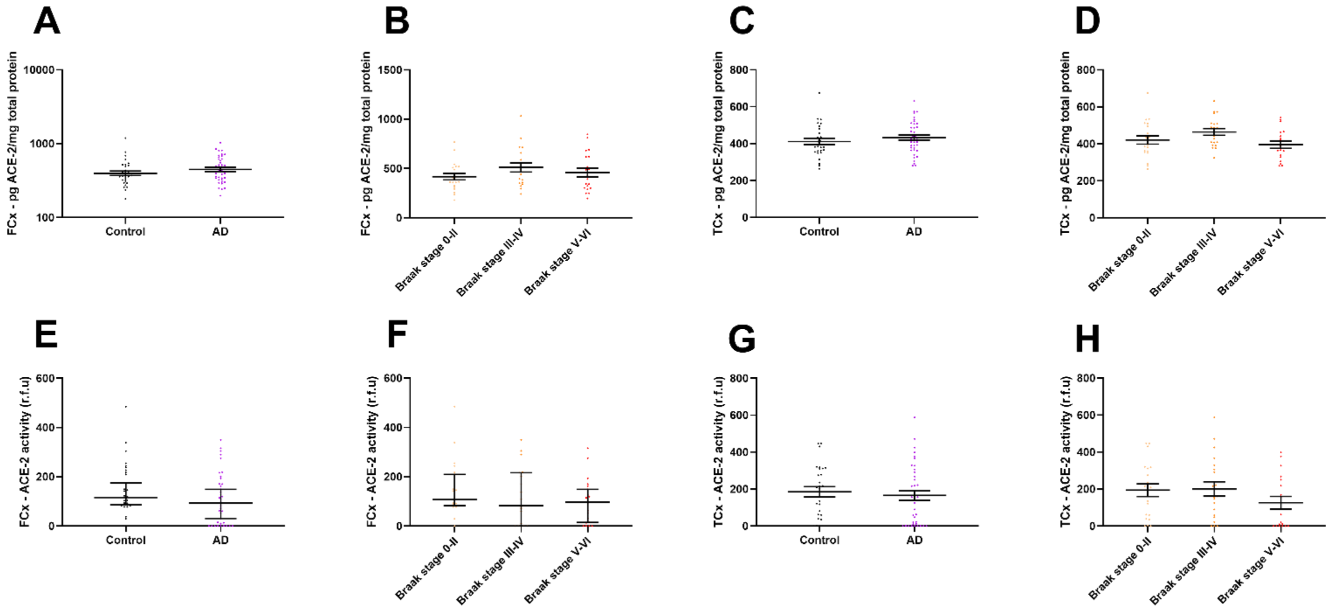

Supplementary Figure 3. ACE-2 protein level and enzyme activity is unaltered in Alzheimer's disease. A-D. Scatterplots showing ACE-2 protein concentration, normalised to the total protein, was unchanged between AD and controls (control=27, AD=39; p=0.2070) and between Braak stages (BS 0-II=18, BS III-IV=18, BS V-VI=19; p=0.2830) in the frontal and temporal cortex in relation to AD (control=27, AD=38; p=0.3677) and Braak tangle stage (BS 0-II=19, BS III-IV=20, BS V-VI=18; p=0.0654). E-H. Scatterplots showing ACE-2 activity was unchanged in AD (control=27, AD=39; p=0.1592) and Braak tangle stage (BS 0-II=18, BS III-IV=18, BS V-VI=19; p=0.3564) in the frontal and temporal cortex in AD (control=27, AD=38; p=0.6119) and Braak tangle stage (BS 0-II=19, BS III-IV=20, BS V-VI=18; p=0.2830). ACE-2 activity is expressed as relative fluorescence units (r.f.u). Standard error bars and median and 95% confidence intervals are shown.
